# Complete Aortomitral Curtain Dehiscence Resulting in Large Pseudoaneurysm 6 Weeks After Aortic Root Replacement

**DOI:** 10.1016/j.jaccas.2024.102762

**Published:** 2024-11-20

**Authors:** Aabha Divya, Christian Guay, Thoralf M. Sundt, Jordan P. Bloom

**Affiliations:** a Division of Cardiac Surgery, Corrigan Minehan Heart Centre, Massachusetts General Hospital, Boston, Massachusetts, USA; b Department of Anesthesia, Critical Care and Pain Medicine, Massachusetts General Hospital, Boston, Massachusetts, USA

## Abstract

This case report presents a unique challenge of complete aortomitral curtain dehiscence and a large pseudoaneurysm 6 weeks post-aortic root replacement in a patient with infective endocarditis. It underscores the importance of meticulous follow-up in patients who have undergone complex aortic surgeries, especially those with infective endocarditis. The patient’s subtle symptoms of occasional dyspnea and lightheadedness highlight the necessity for a comprehensive evaluation and a high index of suspicion. The aortomitral curtain was successfully reconstructed using a bovine pericardial patch, managing the pseudoaneurysm and restoring heart structural integrity. This case also emphasizes the limitations of current diagnostic criteria for infective endocarditis in the presence of intracardiac prosthetic material, and the need for advanced imaging and interdisciplinary consultations to enhance diagnosis and patient management.

## Graphical Abstract

**Figure:**
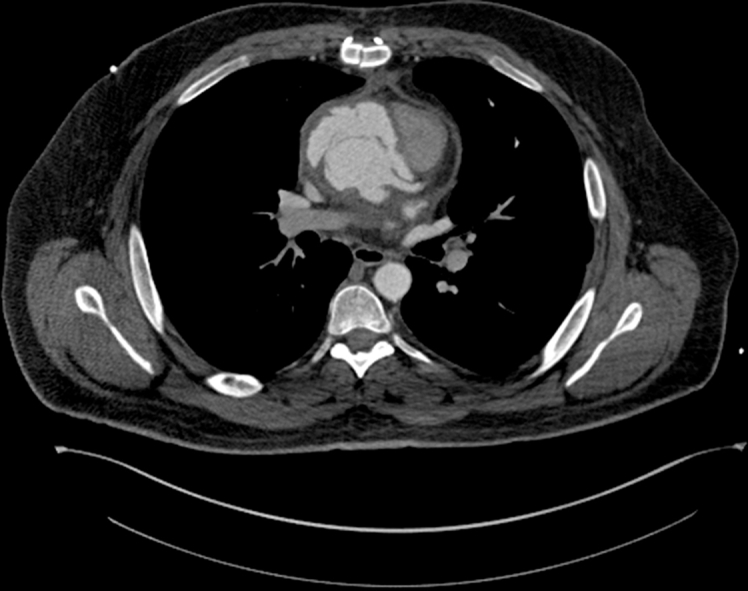

## History of Presentation

A 43-year-old otherwise healthy man originally presented to the hospital with lethargy, fever, and night sweats and was found to have Gemella sanguinis bacteremia and infective endocarditis. Transthoracic echocardiography revealed large aortic valve vegetations with associated valvular destruction and severe aortic regurgitation, as well as circumferential aortic root abscess. He was urgently taken for aortic root replacement with coronary reimplantation and ascending aorta replacement using a 27-mm St. Jude mechanical composite valve. His postoperative recovery was uneventful, and he was discharged home on postoperative day 5.

**Take-Home Messages**

• Early and meticulous follow-up is critical for patients undergoing complex aortic surgeries, especially those with a history of infective endocarditis.
• A thorough examination and a high degree of suspicion should be prompted by subtle symptoms.
• Current diagnostic criteria for infective endocarditis in the presence of intracardiac prosthetic material are limited, emphasizing the importance of advanced imaging and multidisciplinary collaboration in improving patient outcomes.

Approximately 6 weeks later, he presented for routine cardiac surgery follow-up, complaining of occasional dyspnea on exertion and lightheadedness but denied any other symptoms.

## Past Medical History

The patient’s past medical history was significant for hypertension controlled on metoprolol, hyperlipidemia (on statins), prediabetic (glycosylated hemoglobin 5.4%), gastroesophageal reflux disease, and attention deficit hyperkinetic disorder, for which he was on dextroamphetamine-amphetamine.

## Differential Diagnosis

The differential diagnosis for this patient's presentation includes prosthetic valve infective endocarditis, pseudoaneurysm formation, and valvular dehiscence with possible paravalvular leak. Prosthetic valve infective endocarditis should be considered because the patient has a history of infective endocarditis in the presence of prosthetic material. Pseudoaneurysm formation is also possible, particularly given the recent aortic root surgery. Valvular dehiscence with possible paravalvular leak must be considered caused by the patient's previous surgery.

## Investigations

**Figure 1:**
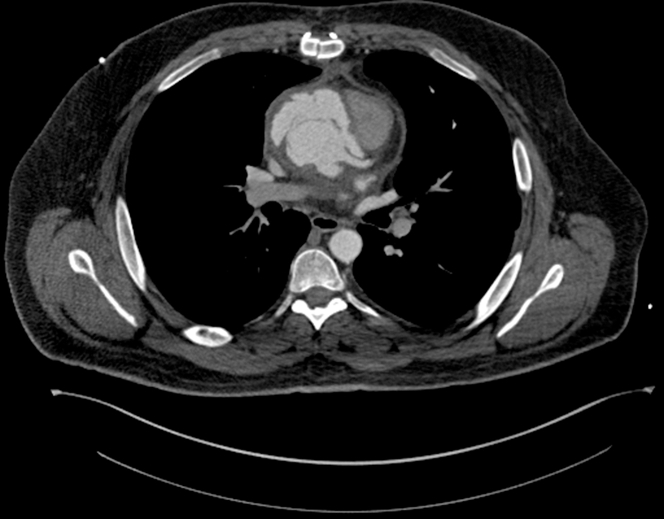
Axial Computed Tomography Chest View Showing Sinuses of Valsalva Pseudoaneurysm axial view of the computed tomography of the chest demonstrating contour abnormality involving the sinuses of Valsalva extending from the aortic root consistent with pseudoaneurysm.

**Figure 2:**
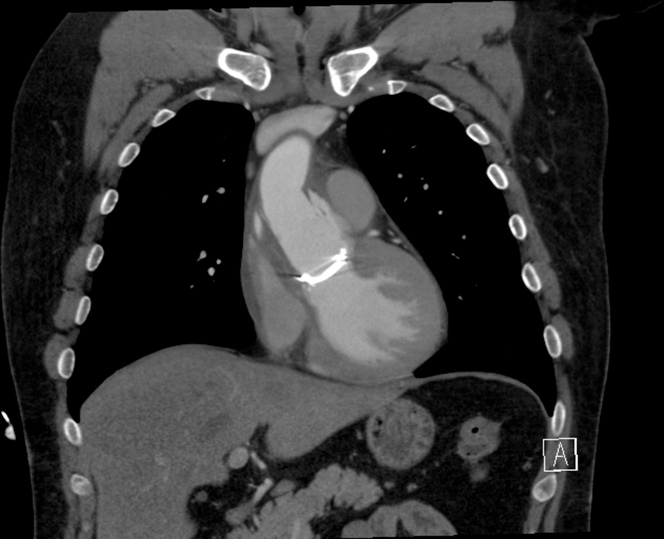
Coronal Computed Tomography Chest View Demonstrating Pseudoaneurysm of the Sinuses of Valsalva Coronal view of the computed tomography of the chest demonstrating contour abnormality involving the sinuses of Valsalva extending from the aortic root consistent with pseudoaneurysm.

**Figure 3:**
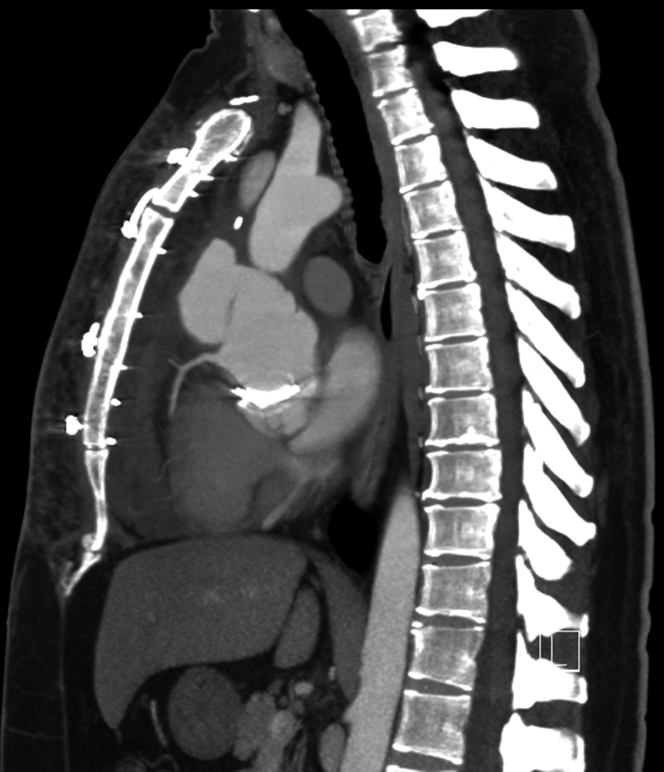
Sagittal Computed Tomography Chest View Illustrating Sinuses of Valsalva Pseudoaneurysm Sagittal view of the computed tomography of the chest demonstrating contour abnormality involving the sinuses of Valsalva extending from the aortic root consistent with pseudoaneurysm.

**Figure 4:**
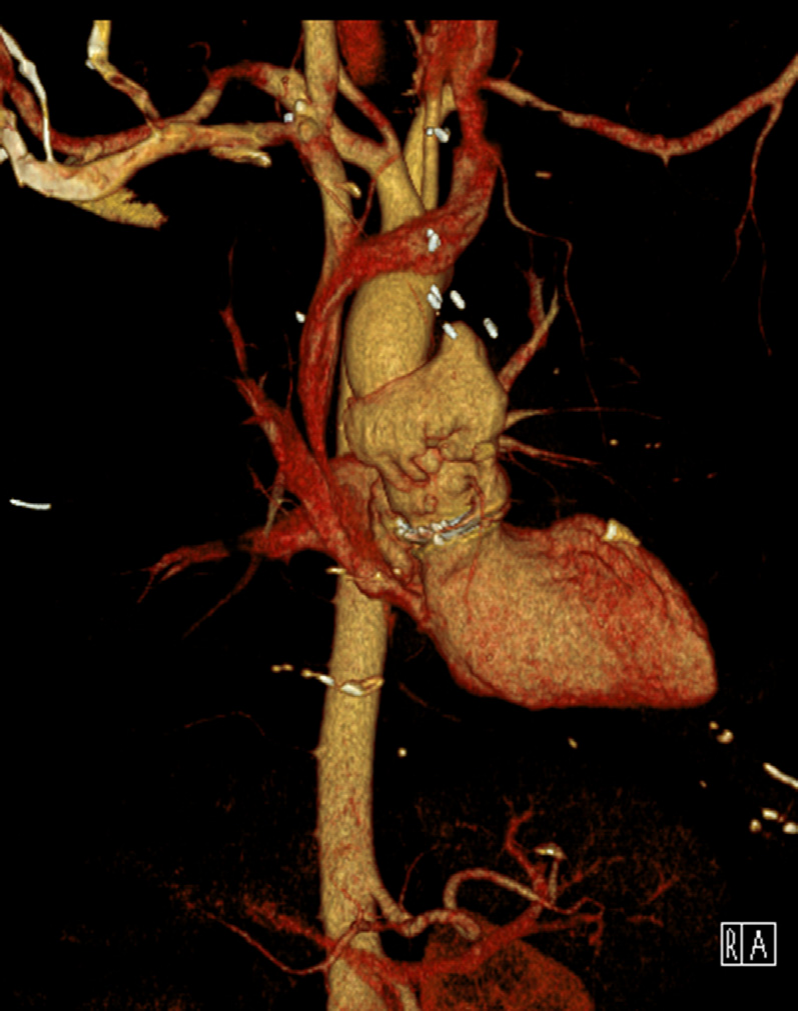
3-Dimensional Electrocardiography-Gated Computed Tomography Reconstruction Highlighting Heart and Aortic Pseudoaneurysm 3-dimensional reconstruction electrocardiography-gated computed tomography of the heart and aorta demonstrating the pseudoaneurysm.

He underwent a chest computed tomography (CT) angiography, which showed extravasation of contrast around the aortic root concerning for large pseudoaneurysm (Figures 1, 2, 3 and 4, Video 1).

## Management

**Figure 5:**
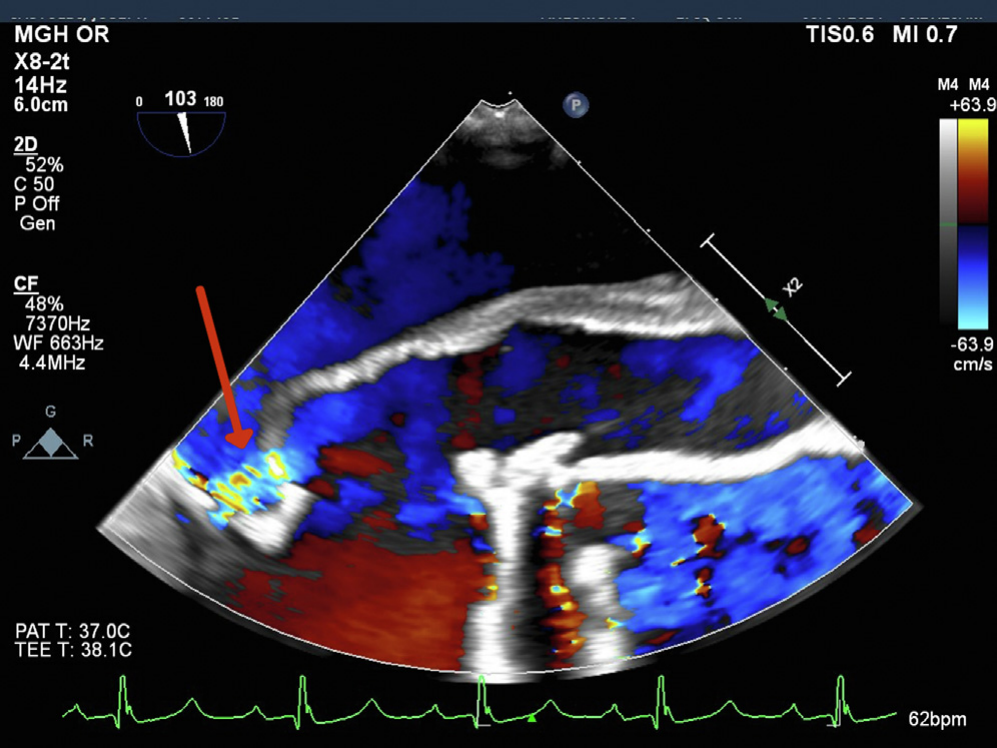
Transesophageal Echocardiogram Displaying Systolic Flow Through Pseudoaneurysm to Left Atrium Transesophageal echocardiogram demonstrating systolic blood flow from the left ventricular outflow tract, through the pseudoaneurysm, and into the left atrium. The red arrow marks the site of the pseudoaneurysm.

**Figure 6:**
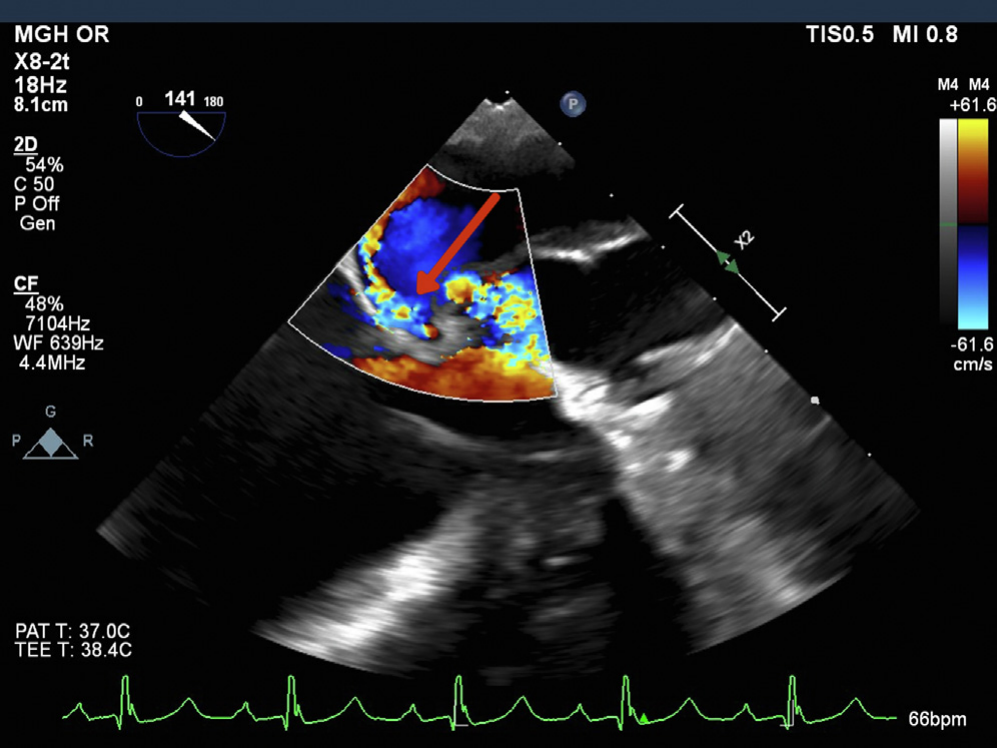
Transesophageal Echocardiogram Demonstrating Systolic Blood Flow via Pseudoaneurysm to Left Atrium Transesophageal echocardiogram demonstrating systolic blood flow from the left ventricular outflow tract, through the pseudoaneurysm, and into the left atrium along the anterior mitral leaflet. The red arrow marks the site of the pseudoaneurysm.

A multidisciplinary preoperative evaluation was conducted, leading to the decision for revision sternotomy and redo aortic root replacement. Induction of general anesthesia and sternal re-entry was uneventful. Transesophageal echocardiography revealed normal biventricular function, mild mitral regurgitation, mildly enlarged aortic sinuses (4.5 cm), a mechanical aortic valve (peak and mean gradients 18 and 11 mm Hg, respectively), absence of the anterior mitral annulus and aortomitral continuity, and a large pseudoaneurysm. During systole, blood flow was noted from the left ventricular outflow tract (LVOT) into the pseudoaneurysm and then into the left atrium. During diastole, blood flow reversed from the pseudoaneurysm back into the LVOT (Figures 5 and 6, Videos 2 to 6).

The pericardial adhesions were very dense, and planes were nonexistent. The large pseudoaneurysm was obvious on re-entry, and efforts were made to avoid dissecting near the area. Cardiopulmonary bypass was initiated by left femoral venous cannulation and central aortic cannulation using the Seldinger technique distal to the pseudoaneurysm to avoid disruption. The proximal aortic arch was dissected beyond the pseudoaneurysm to enable an aortic cross-clamp site. After suspension of cardiopulmonary bypass pump flow, the aorta was clamped, and the heart was arrested in diastole with antegrade and retrograde cardioplegia. The pseudoaneurysm was opened to decompress the heart, and the Valsalva graft was transected to expose the proximal anastomosis. The mechanical valve had been secured with a combination of hand-tied sutures and Cor-Knot crimps, which were removed one by one with extreme care to avoid loss of pledgets. Approximately one-third of the sutures had already dehisced, along the region of the aortomitral continuity. Once the valve and conduit were removed, the LVOT was examined and carefully debrided. There was no aorto-mitral fibrous continuity remaining. The anterior mitral valve leaflet was completely detached from the aortic valve with no visible annulus remaining. A bovine pericardial patch was fashioned and sutured from trigone to trigone directly to the anterior leaflet of the mitral valve and then to aorta. Next, the patch was sewn to the roof of the left atrium, obliterating the dead space of the pseudoaneurysm. Ethibond 2-0 pledgeted valve sutures were placed circumferentially and, where appropriate, were taken through the patch. The sutures were carried through the sewing ring of a 27-/29-mm On-X valved conduit, and all were hand-tied. Next, the coronary buttons were reattached to the graft in their respective sinuses using a 5-0 Prolene suture. Finally, the graft-to-graft anastomosis was performed using a 4-0 Prolene suture. After de-airing and with suspension of pump flow, the cross-clamp was removed. After a period of rewarming and reperfusion, separation from cardiopulmonary bypass was uneventful. Careful examination by transesophageal echocardiography (TEE) confirmed successful reconstruction of the aortomitral curtain, no flow in or out of the pseudoaneurysm cavity, trace mitral regurgitation, and a well-seated mechanical aortic valve in a composite aortic graft without any aortic regurgitation (Videos 7 to 9).

Postoperative transthoracic echocardiography confirmed a well-seated and well-functioning mechanical prosthesis (peak and mean prosthetic gradients of 20 and 10 mm Hg, respectively), a composite graft extending to the ascending aorta, and the bovine pericardial patch at the aorto-mitral curtain, extending from the anterior mitral leaflet to the composite aortic graft. There was residual space at the site of the original aortic pseudoaneurysm without residual flow (Video 10).

## Outcome and Follow-Up

Postoperative recovery was smooth. Echocardiographic and CT imaging confirmed the absence of residual pseudoaneurysm flow and the integrity of the reconstructed aortomitral curtain. The patient was discharged in stable condition.

## Discussion

The sensitivity and specificity of the modified Duke criteria for native valve endocarditis are both suboptimal, at approximately 80%. Diagnostic accuracy for intracardiac prosthetic material-related infection is even lower.^1^ The patient presented with nonspecific symptoms of lightheadedness and occasional dyspnea at the follow-up clinic. In the initial surgery, the patient had undergone a Bentall procedure, which consists of replacing the aortic valve and the aorta with a valve prosthesis and Valsalva graft, respectively, along with reimplantation of the coronary arteries to the graft. After a Bentall procedure, CT is the imaging modality of choice for routine follow-up and to evaluate for complications.^2^ In addition, it provides improved visualization of perivalvular extension of infective endocarditis but less accurate visualization of vegetations compared with TEE. Therefore, CT angiography together with TEE is advisable for optimal management of patients with infective endocarditis, especially for suspected perivalvular extension of infection.^1^

In this particular case, we encountered a complete disjunction of the aorto-mitral curtain coupled with near-total destruction of the anterior mitral annulus and aortic annulus, with a resultant large pseudoaneurysm. This critical situation demanded an innovative approach to reconstruction, which was achieved through the use of a bovine pericardial patch sutured directly to the anterior mitral leaflet. Although there are anecdotal reports addressing the management of such complex issues, each case requires a bespoke solution meticulously tailored to the patient’s specific needs.

## Conclusions

This case highlights the need for early identification of complications following aortic root replacement, especially in patients with a history of infective endocarditis. The surgical repair of complete aortomitral curtain dehiscence with a pseudoaneurysm requires an individualized approach to ensure optimal outcomes.

## Funding Support and Author Disclosures

The authors have reported that they have no relationships relevant to the contents of this paper to disclose.

## Appendix

Video 1 CT Coronal View Video Showing Extent of Pseudoaneurysm From Aortic Root to Sino-Tubular Junction Computed tomography video demonstrating the coronal view of the chest demonstrating the extent of the pseudoaneurysm, extending from the aortic root distally above the sino-tubular junction.

Video 2 Midesophageal Echocardiogram Video of Systolic Flow Through Pseudoaneurysm into Left Atrium Midesophageal echocardiogram demonstrating systolic blood flow from the left ventricular outflow tract through the pseudoaneurysm and into the left atrium.

Video 3 Midesophageal Echocardiogram Video Demonstrating Pseudoaneurysm Flow and Anterior Mitral Valve Motion Midesophageal echocardiogram demonstrating systolic blood flow from the left ventricular outflow tract into the pseudoaneurysm. Also notable is the absence of an anterior mitral annulus and excessive systolic motion of the anterior mitral valve during expansion of the pseudoaneurysm. Only mild mitral regurgitation is noted.

Video 4 Midesophageal Echocardiogram Video Showing Pseudoaneurysm Flow With Absent Anterior Mitral Annulus Midesophageal echocardiogram demonstrating systolic blood flow from the left ventricular outflow tract into the pseudoaneurysm. Also notable is the absence of an anterior mitral annulus and excessive systolic motion of the anterior mitral valve during expansion of the pseudoaneurysm. Only mild mitral regurgitation is noted.

Video 5 Deep Transgastric Echocardiogram Video of Systolic Flow Through Pseudoaneurysm to Left Atrium Deep transgastric echocardiogram demonstrating systolic blood flow from the left ventricular outflow tract through the pseudoaneurysm and into the left atrium.

Video 6 3D Transesophageal Echocardiogram (Surgeon’s View) of Large Pseudoaneurysm Connected to Left Ventricular Outflow Tract Three-dimensional transesophageal echocardiogram of the mitral valve (surgeon’s view) demonstrating a large pseudoaneurysm directly connected to the left ventricular outflow tract.

Video 7 Midesophageal Echocardiogram Video of Patch Repair of Aortomitral Continuity With No Residual Pseudoaneurysm Flow Midesophageal echocardiogram demonstrating patch repair of the aortomitral continuity, with no residual flow in or out of the pseudoaneurysm. Blood in the pseudoaneurysm has begun to coagulate.

Video 8 Deep Transgastric Echocardiogram Video Showing Patch Repair of Aortomitral Continuity Deep transgastric echocardiogram demonstrating patch repair of the aortomitral continuity, with no residual flow in or out of the pseudoaneurysm.

Video 9 3D Transesophageal Echocardiogram (Surgeon’s View) of Aortomitral Continuity Patch Repair Three-dimensional transesophageal echocardiogram of the mitral valve (surgeon’s view) demonstrating patch repair of the aortomitral continuity.

Video 10 Postoperative Coronal CT Chest Image Demonstrating Composite Aortic Root Replacement Postoperative computed tomography image of the chest in coronal view demonstrating the composite aortic root replacement.
